# Antibodies to the RNA-binding protein hnRNP A1 contribute to neurodegeneration in a model of central nervous system autoimmune inflammatory disease

**DOI:** 10.1186/s12974-016-0647-y

**Published:** 2016-07-08

**Authors:** Joshua N. Douglas, Lidia A. Gardner, Hannah E. Salapa, Stephen J. Lalor, Sangmin Lee, Benjamin M. Segal, Paul E. Sawchenko, Michael C. Levin

**Affiliations:** Research Service, VA Medical Center, Memphis, TN USA; Department of Neurology, University of Tennessee Health Science Center, 855 Monroe Avenue, Room 415, Memphis, TN 38163 USA; The Neuroscience Institute, University of Tennessee Health Science Center, Memphis, TN USA; Department of Neurology, University of Michigan Medical School, Ann Arbor, MI USA; Neurology Service, VA Ann Arbor Health Care System, Ann Arbor, MI USA; Laboratory of Neuronal Structure & Function, The Salk Institute, La Jolla, CA USA

**Keywords:** RNA-binding protein, Multiple sclerosis, hnRNP A1, Neurodegeneration, Experimental autoimmune encephalomyelitis

## Abstract

**Background:**

Neurodegeneration is believed to be the primary cause of permanent, long-term disability in patients with multiple sclerosis. The cause of neurodegeneration in multiple sclerosis appears to be multifactorial. One mechanism that has been implicated in the pathogenesis of neurodegeneration in multiple sclerosis is the targeting of neuronal and axonal antigens by autoantibodies. Multiple sclerosis patients develop antibodies to the RNA-binding protein, heterogeneous nuclear ribonucleoprotein A1 (hnRNP A1), which is enriched in neurons. We hypothesized that anti-hnRNP A1 antibodies would contribute to neurodegeneration in an animal model of multiple sclerosis.

**Methods:**

Following induction of experimental autoimmune encephalomyelitis (EAE) by direct immunization with myelin oligodendrocyte glycoprotein, mice were injected with anti-hnRNP A1 or control antibodies. Animals were examined clinically, and the central nervous system (CNS) tissues were tested for neurodegeneration with Fluoro-Jade C, a marker of degenerating neural elements.

**Results:**

Injection of anti-hnRNP A1 antibodies in mice with EAE worsened clinical disease, altered the clinical disease phenotype, and caused neurodegeneration preferentially in the ventral spinocerebellar tract and deep white matter of the cerebellum in the CNS. Neurodegeneration in mice injected with hnRNP A1-M9 antibodies compared to control groups was consistent with “dying back” axonal degeneration.

**Conclusions:**

These data suggest that antibodies to the RNA-binding protein hnRNP A1 contribute to neurodegeneration in immune-mediated disease of the CNS.

## Background

Neurodegeneration, including neuronal and axonal damage, has been shown to contribute to the pathogenesis of multiple sclerosis (MS) [1–6]. Manifestations of neurodegeneration have been observed using neuroradiological, neuropathological, and animal studies of MS. For example, magnetic resonance imaging (MRI) brain images of MS patients show axonal damage as well as brain and spinal cord atrophy, which correlate with neurological disability [7, 8]. Pathologically, accumulation of amyloid precursor protein (APP) [9, 10] and staining for non-phosphorylated neurofilament [3] (both markers of axonal injury) showed that axonal damage is a major component of MS lesions [3, 5, 11, 12]. Importantly, several studies have shown the presence of neurodegeneration throughout the disease course of MS, not just during latter progressive phases of disease, as was originally thought [5]. In experimental autoimmune encephalomyelitis (EAE), an animal model of MS, data indicate that, as in MS, neuronal and axonal damage are present throughout the course of the disease and thus contribute to the neurologic disability [13–15]. Further, axonopathy is a prominent feature of autoimmune demyelinating disease induced by myelin-reactive Th1 or Th17 cells [16].

Antibody targeting of neuronal and axonal antigens is one of the several proposed mechanisms that might underlie neurodegeneration in MS and EAE [17]. For example, MS patients develop antibodies to the “axolemma-enriched fraction”, neurofilaments (NFs), and neurofascin [18–22]. In mice, immunization with NF-L protein resulted in spastic paraparesis concurrent with spinal cord axonal degeneration [22, 23]. The mice developed a pro-inflammatory T cell response and importantly, also developed antibodies to NF-L and IgG deposits within the axons of the spinal cord lesions [22, 23]. Further, MS patients were found to make antibodies to neurofascin [24, 25], a protein with an isoform present on the axons at the nodes of Ranvier [24, 25]. Application of these antibodies to hippocampal slice cultures inhibited axonal conduction [24, 25]. Following induction of EAE with myelin oligodendrocyte glycoprotein (MOG)-specific T cells, the addition of anti-neurofascin antibodies augmented disease [24, 25].

MS patients (in contrast to healthy controls) have also been found to develop antibodies to heterogeneous nuclear ribonucleoprotein A1 (hnRNP A1), a ubiquitously expressed RNA-binding protein (RBP) that is enriched in neurons [11, 12, 17, 26–28]. Importantly, RBPs, including hnRNP A1, regulate RNA metabolism, and dysfunctional RBPs have been shown to cause neurodegeneration in amyotrophic lateral sclerosis (ALS) and frontotemporal lobe dementia (FTLD) [29–31]. The immunodominant epitope of hnRNP A1 recognized by MS IgG was experimentally determined to include “M9”, hnRNP A1’s nucleocytoplasmic shuttling domain, which is required for its transport into and out of the nucleus to the cytoplasm [17, 27, 32, 33]. In an in vitro model of neurodegeneration, anti-M9 antibodies that bind the MS “M9” immunodominant epitope caused neurodegeneration (as shown by Fluoro-Jade C staining), apoptosis, and changes in gene expression related to hnRNP A1 function and the clinical phenotype of MS patients (i.e., spastic paraparesis and ataxia) [11, 17, 27]. Anti-M9 antibodies were found to enter neurons in vitro by utilizing endocytosis, a mechanism identical to antibodies isolated from ALS patients and monoclonal antibodies directed at Tau protein [17, 34–36]. Further, the anti-M9 antibodies caused the mis-localization of hnRNP A1 from predominantly nuclear to an equal nuclear/cytoplasmic distribution suggesting that the anti-M9 antibodies bound M9 and disrupted hnRNP A1’s normal trafficking between the nucleus and cytoplasm [17, 34].

Considering these data, we hypothesized that anti-hnRNP A1-M9 antibodies might promote neurodegeneration in vivo. We tested this hypothesis by injecting anti-hnRNP A1-M9 antibodies in C57BL/6J mice with MOG-induced EAE. MOG-C57BL/6J EAE mice develop chronic disease with very little variation once peak disease has been achieved. Brown and Sawchenko meticulously mapped the timing of neurodegeneration of the entire central nervous system (CNS) using Fluoro-Jade C, a marker of degenerating neural elements [13]. We paralleled this paradigm and found that the anti-M9 antibodies caused neurodegeneration in several neuronal pathways with a strong clinical correlation with signs and symptoms of MS patients.

## Methods

### Animals

C57BL/6J and SJL/J female mice were purchased from Jackson Laboratories (Bar Harbor, ME). The mice were 11 weeks of age at the experimental onset and were housed on a 12:12-h light:dark cycle, with standard chow and water freely available under pathogen-free conditions. A sentinel mouse was present in each room, which was routinely assessed for mouse-borne illnesses. All animal procedures were reviewed and approved by the University of Tennessee and Veterans Affairs Medical Center—Memphis Institutional Animal Care and Use Committees.

### Experimental design, induction of experimental autoimmune encephalomyelitis, and antibody injections

For EAE, the mice were divided into four groups. Group #1 (labeled “control”) consisted of “untouched” mice that did not receive EAE or injections and were housed under the same conditions as those mice that received EAE. Group #2 contained mice that received EAE and injections of phosphate-buffered saline (PBS) intraperitoneally (ip). Group #3 received EAE and mouse IgG2b (isotype control) injections (ip), and group #4 received EAE and mouse IgG2b anti-hnRNP A1 antibodies ip (experimental group). The experimental paradigm was as follows. On day 0, the mice were immunized subcutaneously on the back between both the upper and hind limbs with an epitope of myelin oligodendrocyte glycoprotein (MOG_35–55_; 100 μg/mouse), emulsified in complete Freund’s adjuvant (CFA) containing 400 mg/ml heat-killed *Mycobacterium tuberculosis* (H37Ra; Difco). Pertussis toxin (150 ng) was administered via ip injection on days 0 and 1. Pertussis toxin and MOG_35–55_/CFA were obtained from Hooke Laboratories (cat no. EK-2110) (Lawrence, MA). The mice were checked daily for clinical signs of EAE (see clinical scoring section below). Upon the first clinical signs, the mice were injected ip with either PBS, mouse IgG2b (isotype control) (Millipore MABC006), or mouse anti-hnRNP A1-M9 antibodies (Millipore 04-1469). The epitope of the hnRNP A1-M9 antibodies overlaps with the immunodominant epitope of IgG isolated from MS patients [27, 34]. Each animal received three injections of 100 μg of antibody per injection on days 0, 2, and 4 from the initial signs of EAE.

For antibody localization studies, anti-hnRNP A1-M9 and isotype control IgG antibodies were conjugated with CF680, a near-infrared (nIR) marker, according to the manufacturer’s instructions (Caliper/Perkin Elmer, Waltham). Individual mice were injected with pertussis (200 ng on day 0), followed by the nIR-labeled antibodies (10 mcg once intravenously via tail vein) on day 3. Twenty-four hours later, the animals were sacrificed; CNS tissues were removed and visualized using an in vivo imaging system (IVIS—Perkin Elmer, SpectrumCT In Vivo Imaging System 128201), per the manufacturer’s instructions.

### Clinical scoring

Clinical scoring was adapted from “Appendix A—EAE scoring guide” found in the protocol of the Active Induction EAE Kit from Hooke Laboratories (Hooke cat no. EK-2110). The scoring system is composed of scoring values ranging from 0 to 5.0 increasing at increments of 0.5. The mice were scored daily throughout the study. A score of 1.0 indicates a “limp tail” (an initial sign of clinical disease onset), whereas 5.0 is moribund. The mice with scores of 4.0 or higher for more than 2 days were euthanized and given a score of 5.0 for the remainder of the study. Quantification was performed in GraphPad Prism software. Statistical significance was analyzed by two-way repeated measures analysis of variance (ANOVA) (*p* ≤ 0.05). Bonferroni post-tests were performed when significant differences were indicated. Additionally, a notation of whether “spastic gait” was observed in an individual animal was recorded. Spastic gait was described as abnormally “stiff” or “flexed” extremities, compared to “typical” EAE, when mice develop a “limp” or flaccid paralysis in which their legs drag.

### Tissue preparation and analyses of neurodegeneration by Fluoro-Jade C staining of degenerating neural elements

The mice were either euthanized by perfusion after two consecutive days of a 4.0 or higher score or at the end of the study. Intracardiac perfusion was performed via the aorta. The mice were perfused with 50 ml of ice-cold PBS until fluid ran clear, followed by 250 ml of ice-cold 4 % buffered paraformaldehyde. The brains and spinal cords were extracted and post-fixed in 4 % paraformaldehyde for 48 h at 4 °C. The brain tissues were sectioned at 30 μm utilizing a sliding microtome. The spinal cord tissue was paraffin embedded and sectioned at 10 μm. The sections were stained with hematoxylin and eosin and were used as reference.

Fluoro-Jade C (Millipore AG325) was utilized to label degenerating neural elements in both fixed, frozen brain tissue as well as paraffin-embedded, fixed, spinal cord tissue [13, 37]. Fluoro-Jade C is a fluorescein derivative that selectively stains degenerating neural elements [13, 37]. Fluoro-Jade C histochemistry yields results consistent with other markers of neurodegeneration including staining of non-phosphorylated neurofilament heavy-chain subunits and ultrastructural changes shown by electron microscopy [38]. In EAE, Fluoro-Jade C appears to stain axons and terminal-like structures with a strong correlation with SMI-32 (a marker of non-phosphorylated neurofilament heavy-chain subunits) in white matter tracts [6]. This was also confirmed by ultrastructural analyses by electron microscopy, which showed thinning of myelin sheaths and severely disordered and occasional empty myelin sheaths [6]. Fluoro-Jade C stains neuronal cell soma less frequently and robustly compared to SMI-32 [6]. In this study, the brain and spinal cord sections labeled with Fluoro-Jade C were analyzed while referencing the Interactive Atlas Viewer (atlas.brain-map.org) and Brown and Sawchenko [13], which contained a comprehensive Fluoro-Jade C staining map of the entire CNS in MOG_35–55_-induced EAE. Quantification of Fluoro-Jade C intensity was performed using ImageJ software, which calculated the densitometric mean of fluorescence intensity, using a method adapted from Burgess et al. [39]. Specifically, for each group, 10 high power fields from at least three different animals (*n* = 30 measurements per group) were analyzed. Within each high power field, background fluorescent intensity was subtracted from the mean fluorescent intensity, which resulted in the measurement of degenerating axons within that field. Thirty different fields per group (10 high power fields from at least three animals) were examined, and the mean of these 30 measurements was used to evaluate whether there were differences between the experimental groups using the student’s *t* test.

## Results

### Anti-hnRNP A1-M9 antibodies exacerbate MOG_35–55_-induced EAE

**Fig. 1:**
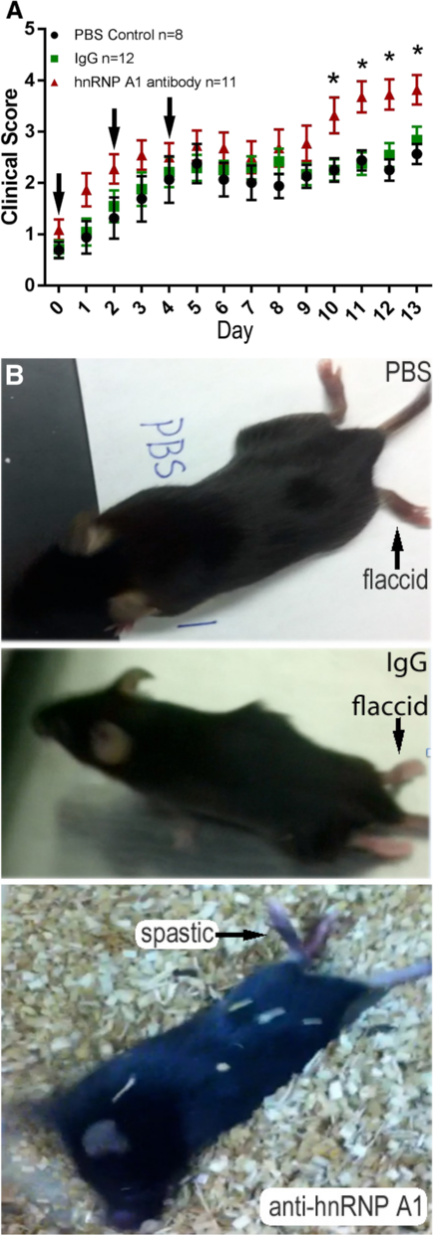
Clinical progression of EAE in anti-hnRNP A1-M9-injected mice compared to controls. **a** EAE was induced in C57BL/6J mice with active immunization using MOG_35–55_. At the first clinical signs of disease (limp tail) (designated as day 0 in this graph), the mice were injected with 100 μg of PBS (*black*), isotype-matched IgG2b (*green*), or anti-hnRNP A1-M9 (*red*) antibodies. Injections were given at days 0, 2, and 4 for a total of three injections (arrows). EAE mice were scored daily from the disease onset using the following standard scale: 1—limp tail; 2—hind limb weakness/wobbly gait; 3—hind limb paralysis; 4—hind limb paralysis/weakness of front limbs; and 5—moribund. Quantification was performed in GraphPad Prism software. Statistical significance was analyzed by two-way repeated measures analysis of variance (ANOVA) (*p* ≤ 0.05). **b** Anti-hnRNP A1-M9 antibody-treated mice developed increased spastic paralysis compared to an isotype control IgG2b and PBS-injected animals, which develop flaccid paralysis

Following induction of EAE, the mice were monitored daily for early clinical signs (limp tail) of EAE. Upon the first clinical signs of EAE (day 0), the mice were injected with PBS (injection control), anti-mouse IgG2b (isotype control), or mouse anti-hnRNP A1-M9 antibodies (100 μg ip on days 0, 2, and 4, for a total of three injections) (Fig. 1). Naïve C57BL/6J mice (non-EAE, no injections) were used as a non-disease control. Anti-hnRNP A1-M9 antibody-treated mice developed statistically worse clinical disease compared to all other groups (Fig. 1a). Interestingly, anti-hnRNP A1-M9-treated mice developed a spastic gait at a higher frequency than the control groups (Fig. 1b, an example). In a typical experiment, 50 % of the anti-hnRNP A1-M9-injected animals were spastic compared to only 10 % of the mice in other EAE groups. Furthermore, several anti-hnRNP A1-M9 mice had to be euthanized prior to the end of the study due to the severity of the spasticity and clinical disease, whereas mice in the other two groups did not commonly reach a score of 4, which indicates a severe disease state. Additionally, in contrast to all other groups, some of the anti-hnRNP A1-M9 mice developed tail lesions (30 % of anti-hnRNP A1 EAE compared to 0 % of PBS or isotype control EAE). Importantly, published studies have associated tail lesions in EAE with increased disease severity and immune responses [37]. Taken together, these data indicate that the anti-M9 antibodies contributed to worse disease and a change in the phenotype of the mice (spastic compared to flaccid paralysis).

### Anti-hnRNP A1-M9 antibodies cause increased levels of neurodegeneration of specific CNS pathways in EAE

After observing that anti-hnRNP A1-M9 antibodies worsened EAE, we hypothesized that the augmented clinical progression might be related to changes in neurodegeneration in the CNS. To examine this, we stained the brains and spinal cords of three animals from each group with Fluoro-Jade C, a marker of degenerating neural elements. Fluoro-Jade C has been used previously to map neurodegeneration in the identical active MOG_35–55_-induced EAE protocol utilized in these studies. We compared the data between the groups and used the map of Fluoro-Jade C neurodegeneration in EAE published by Brown and Sawchenko as reference [13].

**Fig. 2:**
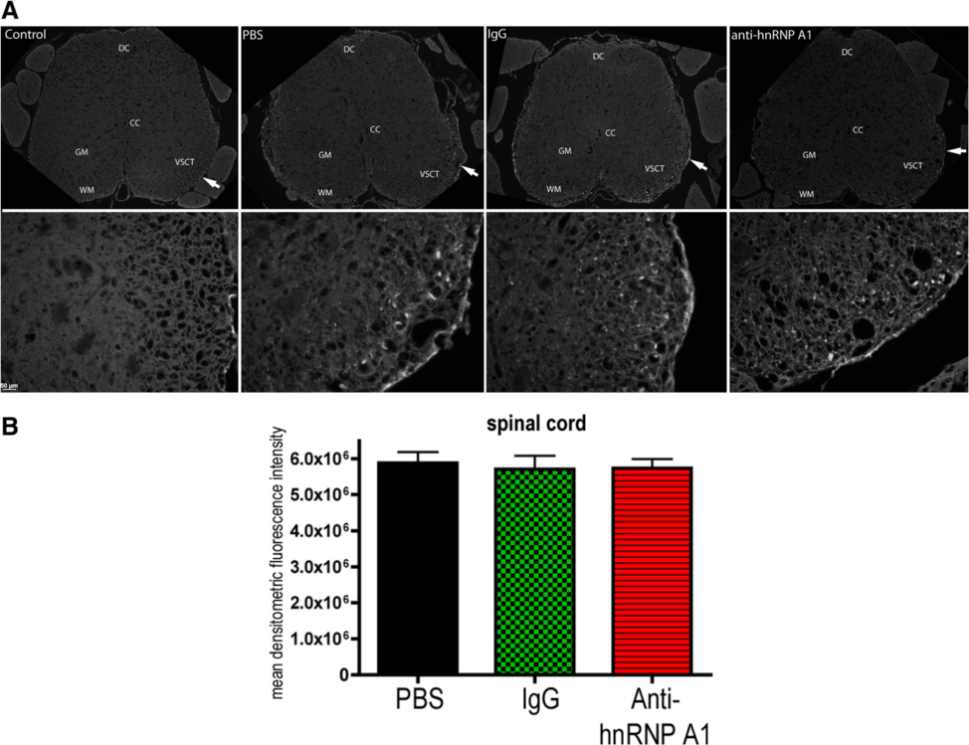
Fluoro-Jade C staining of the spinal cord. **a**. Paraffin-embedded sections from the lumbosacral region of the spinal cord were sectioned at 10 μm. The sections were stained for neurodegeneration using the Fluoro-Jade C. There was no staining of neural elements in control (non-EAE) mice. At low power, the spinal cords from all three EAE groups showed staining that was greater in the white matter tracts than in the gray matter. High-powered images were taken from representative areas (shown by *arrows* in the low power view) of the ventral spinocerebellar tract (VSCT), showing similar levels of Fluoro-Jade C staining. **b** Quantification of Fluoro-Jade C intensity was performed on 10 images from three animals per experimental group (30 images per condition). The mean densitometric fluorescence intensity was calculated using ImageJ software, and groups were compared using the student’s *t* test. There were no differences in fluorescence intensity in the spinal cords of the three EAE groups. Abbreviations: *PBS* EAE mice injected with PBS, *IgG* EAE mice injected with isotype control IgG2b, *Anti-hnRNP A1* EAE mice injected with anti-hnRNP A1-M9 antibodies, *DC* dorsal columns, *CC* central canal, *GM* central gray matter, *WM* white matter, *VSCT* ventral spinocerebellar tract

**Fig. 3:**
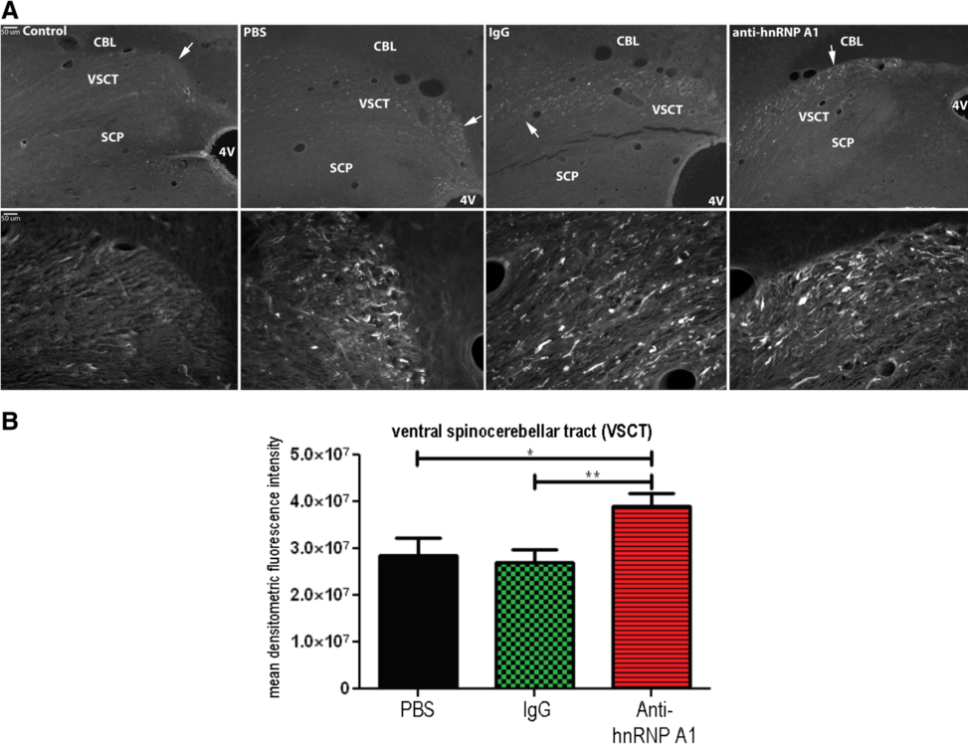
Fluoro-Jade C staining of the ventral spinocerebellar tract of the brainstem. **a** Fixed, frozen 30-μm brainstem (dorsal medulla) sections were stained with Fluoro-Jade C. Low-powered images show staining of the ventral spinocerebellar tract (*VSCT*) with no staining of the superior cerebellar peduncle (*SCP*). High-powered images are taken from the area of the VSCT labeled with *arrows*. **b**. Quantification of Fluoro-Jade C intensity was performed on 10 images from three animals per experimental group (30 images per condition). The mean densitometric fluorescence intensity was calculated using ImageJ software, and groups were compared using the student’s *t* test. *p ≤ 0.05; **p ≤ 0.01. *CBL* cerebellum, *4V* 4th ventricle

**Fig. 4:**
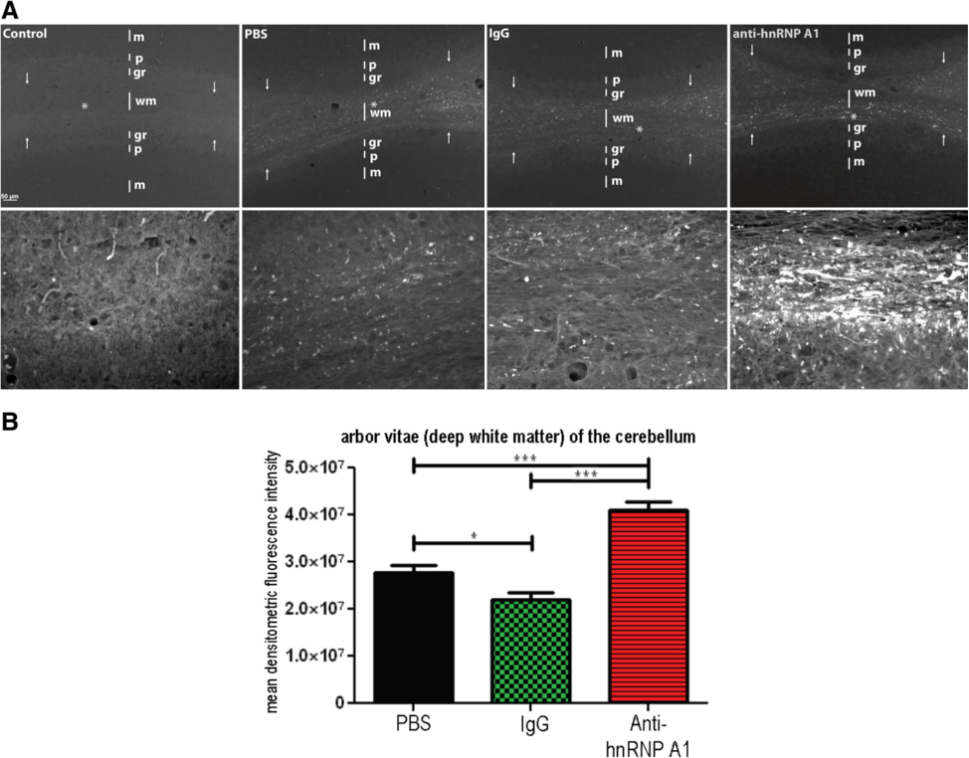
Fluoro-Jade C staining of the deep cerebellar white matter (“arbor vitae”). **a** Fixed, frozen 30-μm sections from the cerebellum were stained with Fluoro-jade C. Low-powered images show intense staining of the deep cerebellar white matter (*wm*) with minimal punctate staining of the granule layer (*gr*). There was no staining of Purkinje cells (*p*) or within the molecular layer (*m*). High-powered images were photographed from within the white matter (*wm*) labeled with an *asterisk. Arrows* represent the borders of the deep white matter of the cerebellum. **b** Quantification of Fluoro-Jade C intensity was performed on 10 images from three animals per experimental group (30 images per condition). The mean densitometric fluorescence intensity was calculated using ImageJ software, and groups were compared using the student’s *t* test. *p ≤ 0.05; ***p ≤0.001

There was very minimal background Fluoro-Jade C staining of non-neural elements in the spinal cords or brains of unimmunized (“naïve”, “non-EAE”) animals as previously described (Figs. 2, 3, and 4, “control”) [13]. In the spinal cords of the three EAE groups (PBS, isotype control IgG, anti-hnRNP A1-M9), we found a similar distribution of staining (Fig. 2a). Staining dominated in the ventral spinocerebellar tracts (VSCT) at all levels of the spinal cord, with lesser amounts in the dorsal spinocerebellar tracts (DSCT). There was also a modest degree of staining in the dorsal columns (DC) and throughout the grey matter (GM) of the spinal cord (Fig. 2a). In the spinal cord, there were no differences in the mean densitometric Fluoro-Jade C fluorescence intensity between the three EAE groups (VSCT as an example, Fig. 2b).

Examination of the brainstems of these animals revealed neurodegeneration in the VSCT, trapezoid body, spinal tract of the trigeminal nerve, inferior cerebellar peduncle, and other structures within the brainstem as described previously [13] (Fig. 3a). There was an absence of staining in the superior cerebellar peduncle (SCP). Neurodegeneration was detected in the cerebral peduncles that continued to include the pyramids (not shown) in all EAE-induced animals. The cerebral peduncle data parallels that of Brown and Sawchenko [13], but the pyramidal data differs in that there was no reported Fluoro-Jade C staining in the standard MOG-induced EAE. Both areas involve the corticospinal system, and these differences may be related to the length of the experiments (which were about a week longer in the present experiments). By comparing the neurodegeneration in the brainstems of the three EAE groups, it was determined that there was an increase in Fluoro-Jade C staining in one specific tract, the VSCT, of the anti-hnRNP A1-M9 antibody-treated mice compared to the other EAE mice. The distal VSCT of the brainstem as it entered the cerebellum parallel to the SCP showed increased neurodegeneration in anti-hnRNP A1-M9-treated mice (Fig. 3a). Quantitative analysis (10 images from each of the three mice per group) of the mean densitometric Fluoro-Jade C fluorescence intensity using ImageJ software confirmed an increase in neurodegeneration in the anti-hnRNP A1-M9 mice compared to all other groups (Fig. 3b). The cells of origin of the VSCT are admixed with other neurons within laminae VII, VIII, and IX of the gray matter of the lumbosacral spinal cord [40–45]. Some of the neurons are grouped into partially defined nuclei within layer VII including spinal border cells (SBCs), and the lumbar and sacral pre-cerebellar nuclei [44, 46]. Axons from these neurons immediately cross in the spinal cord at their segment of origin in the ventral white matter. These projections make up the VSCT, which continues up the spinal column through the lateral funiculi, followed by the brainstem and then enters the cerebellum with the SCP, where its projections then enter the deep white matter of the cerebellum [40–45]. The VSCT is the only afferent tract of the SCP. With this in mind, we next examined the cerebellum for changes in neurodegeneration between the three EAE groups.

We observed large quantities of neurodegeneration in the deep white matter (arbor vitae) of the cerebellum (Fig. 4). This area contained some of the most intense Fluoro-Jade C staining of the CNS in EAE [13]. Furthermore, in concordance with the data presented by Brown and Sawchenko, we also saw a lack of neurodegeneration in efferent pathways of the cerebellum including Purkinje cells and the interposed, fastigial, and dentate nuclei [13]. Comparison of all EAE groups as well as naive controls revealed that there was increased neurodegeneration in the deep white matter (arbor vitae) of the cerebellum of anti-hnRNP A1-M9 antibody-treated animals compared to all other EAE groups (Fig. 4a). This finding was quantified and confirmed using ImageJ software, which showed a statistically significant increase in the mean densitometric Fluoro-Jade C fluorescence intensity in the anti-hnRNP A1-M9 group (Fig. 4b). There was minimal staining of the granule layer in the cerebellum, none of which included cell bodies. After investigating the brainstem and the cerebellum, we examined the cerebral cortex for changes in neurodegeneration. We observed identical patterns of neurodegeneration as published previously [13].

### Gross localization of anti-hnRNP A1-M9 and isotype control IgG antibodies in mice

**Fig. 5:**
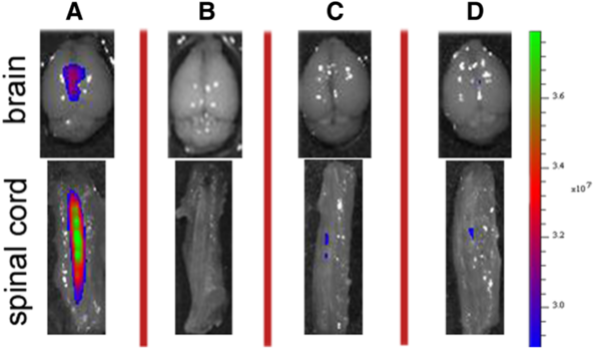
Localization of anti-hnRNP A1-M9 antibodies and isotype control IgG antibodies in the CNS of mice. Following pertussis injection to disrupt the blood brain/spinal cord barrier and injection of near IR-labeled anti-hnRNP A1-M9 antibodies, there is a signal consistent with localization of the antibodies to the brain and spinal cord (**a**). Under identical conditions, there was a minimal signal using the isotype control IgG (**b**). Neither antibody localized to CNS structures without pertussis injection (**c**, **d**)

To begin to examine how anti-hnRNP A1-M9 antibodies might enter the CNS, we injected mice with nIR-labelled antibodies and visualized their localization by IVIS (see the “Methods” section). Intravenous injection with either antibody showed a minimal signal within the CNS structures (Fig. 5c, d). Next, we injected the mice with pertussis toxin to disrupt the blood brain/spinal cord barrier, followed by intravenous injection with either anti-hnRNP A1-M9 or the isotype control IgG antibodies. In this experiment, the anti-hnRNP A1-M9 antibodies localized to both the brain and spinal cord, with the greater signal concentrated in the spinal cord (Fig. 5a). In contrast, the isotype control IgG did not localize to the CNS structures (Fig. 5b).

## Discussion

These data indicate that in EAE, an animal model of MS, antibodies to the “M9” sequence of hnRNP A1 (compared to PBS and isotype-matched IgG2b controls) caused the following: (1) worse clinical disease, (2) an altered clinical phenotype (spastic paraparesis), and (3) increased levels of neurodegeneration in the distal projection of the VSCT and the deep white matter of the cerebellum. Importantly, antibodies to the CNS targets have been shown to contribute to neurodegeneration in MS and EAE [17]. For example, in addition to antibodies to hnRNP A1-M9, MS patients develop antibodies to NFs and neurofascin [17, 22, 24, 25]. In mice, immunization with NF-L caused antibody formation, IgG deposition on axons, and spastic paraparesis concurrent with the spinal cord axonal degeneration [22, 23, 47]. In separate experiments, the addition of anti-neurofascin antibodies following induction of EAE with MOG-specific T cells augmented disease and showed increased APP expression [24]. Like antibodies to NF and neurofascin, the data presented here indicate that antibodies to hnRNP A1-M9 contribute to neurodegeneration in EAE and because these antibodies are present in MS patients, strongly support their role in neurodegeneration in MS [11, 12, 17, 26–28].

Neurodegeneration is now believed to be the primary cause of permanent, long-term disability in MS patients [5, 6, 11, 12]. The brains and spinal cords of MS patients show evidence of both neuronal and axonal degeneration. For instance, there is neuronal loss and apoptosis of cortical pyramidal neurons [48]. In addition, MS brains display markers of axonal degeneration including axonal dystrophy, axonal spheroids, and staining for non-phosphorylated neurofilament (SMI-32) [1–3, 9, 49]. These markers confirm the presence of axonal degeneration in MS but do not implicate a specific mechanism [50]. The cause of neurodegeneration appears to be multifactorial. In MS, there is evidence of both “Wallerian Degeneration”—transection of axons that disconnects the neuronal cell body from its distal axon and “dying back” axonopathy—length-dependent, distal to proximal, progressive axonal degeneration, which occurs in toxic, metabolic, and neurodegenerative diseases [6, 51–53]. Both involve abnormalities in axonal transport demonstrated by the accumulation of APP in axons of MS brains, which occurs in the setting of dysfunctional fast axonal transport [1, 9, 50]. In animal models, axonal degeneration results from a specific sequence of events, which include impaired axonal transport, mitochondrial dysfunction, and increase in intra-axoplasmic calcium [53, 54]. Importantly, each of these abnormalities has been found in brains of MS patients as well as in EAE and have been implicated in the pathogenesis of neurodegeneration.

**Fig. 6:**
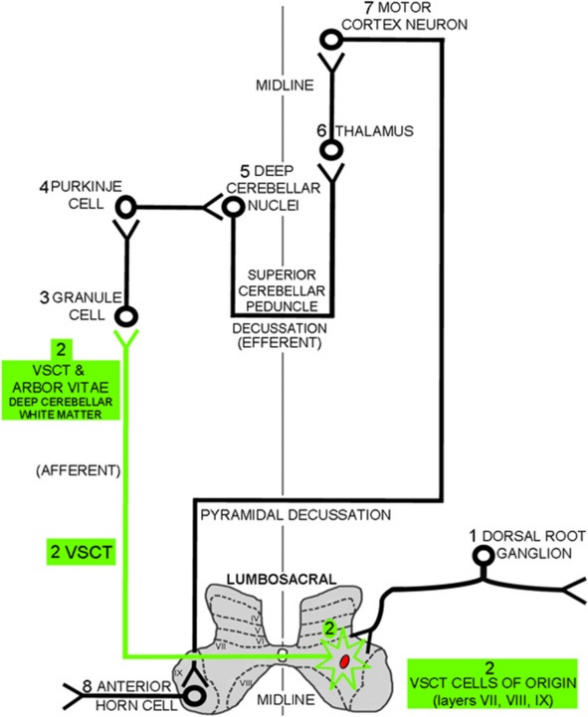
Schematic of the anatomical pathways that involve the VSCT. Afferent input from the peripheral nervous system via the (*1*) dorsal root ganglia synapse on the cells of origins (neuronal cell bodies) of the (*2*) VSCT, which are contained within laminae VII, VIII, and IX of lumbosacral levels of the spinal gray matter. Axons of the (*2*) VSCT, which are preferentially damaged by the anti-hnRNP A1-M9 antibodies (shown in *green*), immediately cross the mid-line of the spinal cord. The (*2*) VSCT then passes through the brainstem where it enters the cerebellum parallel to the superior cerebellar peduncle. (*2*) VSCT axons then pass through the deep cerebellar white matter to synapse on (*3*) granule cells. (*3*) Granule cells synapse on (*4*) Purkinje cells, which in turn, synapse in (*5*) the deep cerebellar nuclei. The (*5*) deep cerebellar nuclei send efferent axons via the superior cerebellar peduncle to the (*6*) thalamus, which relays information to the (*7*) motor cortex, which in turns sends axons that synapse on (*8*) anterior horn cells in the ventral gray of the spinal cord. The (*2*) VSCT (*green*) showed neurodegeneration from the spinal cord up through to its distal axons in the deep white matter of the cerebellum, where it was preferentially affected by the anti-hnRNP A1-M9 antibodies consistent with “dying back” axonal degeneration

Neuroanatomically, we believe the results of these experiments suggest an antibody-mediated autoimmune response to a CNS target that contributes to “dying back” axonal degeneration [6, 50, 53, 55]. We examined the VSCT from caudal to rostral segments starting in the spinal cord and moving to the brainstem and the cerebellum. Specifically, axonal degeneration within the proximal VSCT in the spinal cord was present in both control and anti-hnRNP A1-M9 mice. The cells of origin of the VSCT are contained within laminae VII, VIII, and IX of the lumbosacral spinal cord; thus, the most proximal segments of the VSCT are located in the spinal cord [40–45]. Moving rostrally, neurodegeneration of the VSCT continued in both control and anti-hnRNP A1-M9 mice in the brainstem, but again there were no differences between the groups. As the VSCT entered the cerebellum and continued in the arbor vitae (deep cerebellar white matter), the anti-hnRNP A1-M9 mice showed significantly worse neurodegeneration than either of the control groups. These segments of the VSCT contain its most distal axons as they synapse on granule cells in the cerebellum. Considering there are no differences in proximal, but profound differences in distal segments of the VSCT, this is consistent with “dying back” axonal degeneration (Fig. 5) [6, 50, 53, 55]. Importantly, the VSCT makes afferent connections to the corticospinal tract via cerebellar efferents (Fig. 6). Considering that both cerebellar lesions and genetic mutations in diseases such as the inherited spastic ataxias cause changes in muscle tone [56–58], our data suggest that neurodegeneration of the VSCT might also contribute to spasticity, which was readily apparent in the anti-hnRNP A1 antibody-injected mice.

Although we believe this to be accurate neuroanatomically, this study is limited in that (1) we utilized Fluoro-Jade C, a marker of neurodegeneration that preferentially labels degenerating neural processes compared to neuronal cell bodies (see the “Methods” section), (2) an exact mechanism explaining this observation is not yet apparent and requires further study, and (3) immune markers in this model have not yet been examined. Recent data suggest that dying back axonopathy and Wallerian degeneration might involve a common pathogenic mechanism. This was discovered using the mutant slow Wallerian degeneration (*Wld*^*s*^) mouse [53, 59]. Following nerve injury in these mice, axonal degeneration was delayed compared to wild-type controls. The WLD^s^ protein was isolated and found to be a gain of function mutation [59]. When *Wld*^*s*^ mice were subjected to MOG-induced EAE, neurodegeneration was delayed [59, 60]. Further, when the *Wld*^*s*^ mouse was crossed with mouse models of dying back axonopathy in the peripheral nervous system (PNS) (progressive motor neuropathy—Tbce mutation [61] or the Charcot Marie Tooth (CMT) (type 1B)—MPZ null mutation [62]), degeneration was delayed [59]. In contrast, there was no effect when crossing *Wld*^s^ with *Plp null* mice. The *Plp null* mouse is a model of hereditary spastic paraparesis (HSP) [59, 63], a CNS disorder characterized by dying back axonopathy. These data suggest that more than one mechanism may be involved in dying back axonopathy (i.e., PNS vs. CNS). HSP is clinically and pathologically similar to progressive forms of MS. Interestingly, anti-hnRNP A1-M9 antibodies caused neurodegeneration and altered spinal paraplegia genes (SPG) genes (which when mutated cause HSP) in an in vitro model of neurodegeneration [27].

The mechanism that might cause dying back axonopathy in our model of antibody-mediated neurodegeneration is not yet defined. We believe these data suggest that the site of attack of the anti-hnRNP A1-M9 antibodies might be the cells of origins (neuronal cell soma) of the VSCT, which are present in the lumbosacral spinal cord. Interestingly, anti-hnRNP A1-M9 antibodies localized to the CNS following pertussis injection, suggesting an entry point for the antibodies (Fig. 5). In further support of this hypothesis are two recent studies in EAE. The first showed that the VSCT is one of several pathways that is preferentially targeted by T cells and activated microglia early in the course of EAE concurrent with neurodegeneration [13]. The second showed that entry of pathogenic Th1 and Th17 CD4^+^ T lymphocytes occurs at the L5 lumbar level and that the damage present in EAE is initiated at this level of the CNS [64, 65]. It is possible that both control and anti-hnRNP A1-M9 antibodies enter the CNS at L5 of the spinal cord and that the anti-hnRNP A1-M9 antibodies specifically bind to the VSCT cells of origin (neuronal cell bodies). hnRNP A1 is enriched in large neurons (which contain long axons, like the VSCT) compared to smaller neurons and glia [28, 66]. Thus, in the milieu of a pro-inflammatory immune response, which would allow entry of antibodies into damaged neurons and axons, anti-hnRNP A1 antibodies could bind hnRNP A1, causing it to become dysfunctional. Future in vivo antibody tracing experiment should address the localization of antibodies in EAE. Interestingly, recent data in our lab using an in vitro model of neurodegeneration showed that anti-hnRNP A1-M9 antibodies entered neurons and cause mis-localization of hnRNP A1 to the cytoplasm where it co-localized with stress granules and altered RNA metabolism of spastin [17, 34, 67]. Changes in spastin metabolism cause spasticity [27, 68], a clinical feature of the animals in this study. Whether or not spasticity is a result of dying VSCT axons might not be entirely clear; however, the results of this study unveil a possible explanation for the link between neuronal degeneration and spasticity. Future studies are required to evaluate details of anti-hnRNP A1-M9 antibodies’ involvement in dying back axonopathy, Wallerian degeneration, or other mechanisms of neurodegeneration in autoimmune inflammatory disease of the CNS.

## Conclusions

Anti-hnRNP A1 antibodies contributed to neurodegeneration in an animal model of MS, EAE. Specifically, animals that received the anti-hnRNP A1 antibodies showed worse clinical disease and a change in clinical phenotype from flaccid to spastic paralysis. Further, specific CNS pathways including the VSCT and the deep white matter of the cerebellum underwent preferential neurodegeneration. The pattern of neurodegeneration was consistent with “dying back” axonopathy, one of the several mechanisms involved in the pathogenesis of neurodegeneration in MS. These data suggest that antibodies to the non-myelin protein, hnRNP A1, contribute to neurodegeneration in autoimmune inflammatory disease of the CNS. (Fig. 6 may be used here.)

## Abbreviations

4V, 4th ventricle; ALS, amyotrophic lateral sclerosis; ANOVA, analysis of variance; APP, amyloid precursor protein; C, centigrade; CC, central canal; CFA, complete Freund’s adjuvant; CMT, Charcot Marie Tooth; CNS, central nervous system; DC, dorsal columns; DNA, deoxyribonucleic acid; DSCT, dorsal spinocerebellar tract; EAE, experimental autoimmune encephalomyelitis; FTLD, frontotemporal lobe dementia; GM, grey matter; hnRNP A1, heterogeneous nuclear ribonucleoprotein A1; HSP, hereditary spastic paraparesis; IgG, immunoglobulin G; ip, intraperitoneally; IVIS, in vivo imaging system; ml, milliliter; MOG, myelin oligodendrocyte glycoprotein; MRI, magnetic resonance imaging; MS, multiple sclerosis; NF, neurofilament; ng, nanogram; nIR, near infrared; PBS, phosphate buffered saline; PlP, proteolipid protein; PNS, peripheral nervous system; RBP, RNA-binding protein; RNA, ribonucleic acid; SBC, spinal border cells; SCP, superior cerebellar peduncle; SPG, spinal paraplegia gene; Th, T lymphocyte helper; μg, microgram; μm, micrometer; VSCT, ventral spinocerebellar tract; Wld, Wallerian degeneration mouse; WM, white matter.
